# Paraoxonase 2 modulates a proapoptotic function in LS174T cells in response to quorum sensing molecule N-(3-oxododecanoyl)-L-homoserine lactone

**DOI:** 10.1038/srep28778

**Published:** 2016-07-01

**Authors:** Shiyu Tao, Yanwen Luo, Bin He, Jie Liu, Xi Qian, Yingdong Ni, Ruqian Zhao

**Affiliations:** 1 Key Laboratory of Animal Physiology & Biochemistry, Ministry of Agriculture, Nanjing Agricultural University, Nanjing, Jiangsu, China; 2 Department of Pathology and Laboratory Medicine, University of Vermont Medical Center, Burlington, VT 05452, USA.

## Abstract

A mucus layer coats the gastrointestinal tract and serves as the first line of intestinal defense against infection. N-acyl-homoserine lactone (AHL) quorum-sensing molecules produced by gram-negative bacteria in the gut can influence the homeostasis of intestinal epithelium. In this study, we investigated the effects of two representative long- and short-chain AHLs, N-3-(oxododecanoyl)-homoserine lactone (C12-HSL) and N-butyryl homoserine lactone (C4-HSL), on cell viability and mucus secretion in LS174T cells. C12-HSL but not C4-HSL significantly decreased cell viability by inducing mitochondrial dysfunction and activating cell apoptosis which led to a decrease in mucin expression. Pretreatment with lipid raft disruptor (Methyl-β-cyclodextrin, MβCD) and oxidative stress inhibitor (N-acetyl-L-cysteine, NAC) slightly rescued the viability of cells damaged by C12-HSL exposure, while the paraoxonase 2 (PON2) inhibitor (Triazolo[4,3-*a*]quinolone, TQ416) significantly affected recovering cells viability and mucin secretion. When LS174T cells were treated with C12-HSL and TQ416 simultaneously, TQ416 showed the maximal positive effect on cells viability. However, if cells were first treated with C12-HSL for 40 mins, and then TQ46 was added, the TQ416 had no effect on cell viability. These results suggest that the C12-HSL-acid process acts at an early step to activate apoptosis as part of C12-HSL’s effect on intestinal mucus barrier function.

The gut epithelium is coated with a thick mucus layer that functions as the first-line defensive barrier against invading microbes and pathogenic antigens^1^,^2^. The mucus layer is composed of mucins (MUCs), digestive enzymes, antimicrobial peptides, and immunoglobulins^3^,^4^. MUCs are produced and secreted by goblet cells distributed throughout the entire intestinal tract. The mucus of the large intestine is comprised of two structurally distinct layers: an epithelium-attached inner layer that is nearly sterile and prevents bacteria from entering the epithelium, and an outer unattached layer that is loose and contains some microbiota^5^,^6^,^7^. Both layers are primarily formed by MUC2 mucin, and abnormalities in MUCs expression have been demonstrated for several diseases^8^,^9^,^10^. MUCs are secreted both constitutively and in response to various stimuli including microbial products, hormones, signaling mediator, and filtrating bacteria^11^,^12^.

Bacteria communicate by secreting and sensing small chemical molecules called autoinducers in a process known as quorum sensing (QS). QS is an intercellular signaling mechanism that is thought to allow bacteria to coordinate behaviors at the population level^13^. Many gram-negative bacteria use N-(3-oxododecanoyl)-homoserine lactone (C12-HSL), a small lipid-soluble and membrane-permeant molecule as an autoinducer of QS^14^. In addition to providing a way for bacteria to assess population size and modulate the gene expression of virulence factors, C12-HSL can also affect the functions of the host cells^15^. Due to its lipophilicity, C12-HSL can rapidly enter mammalian cells^16^ can can trigger apoptosis in multiple cell types^17^,^18^,^19^,^20^,^21^,^22^. However, the ability of C12-HSL to induce apoptosis in epithelial cells may be cell-specific. For example, C12-HSL was found to trigger apoptosis in mammary epithelial cells^19^, but not in the liver Hep2 cell line or the lung epithelial cell line CCL185^18^. Varied responses were also observed in the modulation of the expression of proinflammatory factors. Some reports found that C12-HSL increases expression of cytokines such as IL8^23^,^24^, but other studies would that C12-HSL decreased proinflammatory mediators^25^,^26^,^27^,^28^. Although the mechanism mediating these responses remains unknown, the inconsistent results may be explained if the biological effects of C12-HSL on host cells are cell-type specific.

As QS molecules, N-acylhomoserine lactone (AHL) that is produced by gram-negative bacteria in the gut can influence homeostasis of the host intestinal epithelium. This can perturb epithelial integrity and the development of intestinal diseases^29^. Recently, studies have reported detrimental effects of AHLs on intestinal epithelial barrier function and inflammation^23^,^30^,^31^. As the major epithelial barrier on the gut surface, MUCs secreted by goblet cells play an essential role on the maintenance of epithelial homeostasis. However, the effects of AHLs on goblet cells function remains unclear. We report for the first time that C12-HSL but not C4-HSL markedly decreases cell viability and induces apoptosis in a dose- and time-dependent manner in the goblet cell line LS174T. Compared to the slight rescue effects exhibited by lipid-raft disruptor MβCD or oxidative stress inhibitor NAC on cells damage induced by C12-HSL, the paraoxonase 2 (PON2) inhibitor TQ416 can almost completely rescue cell viability and apoptosis of LS174T. Our results indicate that PON2 is a major component mediating C12-HSL-induced apoptotic effects on LS174T cells. These findings will guide our understanding of the underlying causes of intestinal mucus barrier disorder in *Pseudomonas aeruginosa (Pa*) infection patients, and may suggest novel therapeutic targets exploited to limit the pathogenicity of *Pa*.

## Results

### C12-HSL but not C4-HSL decreases cell viability of LS174T cells

**Figure 1:**
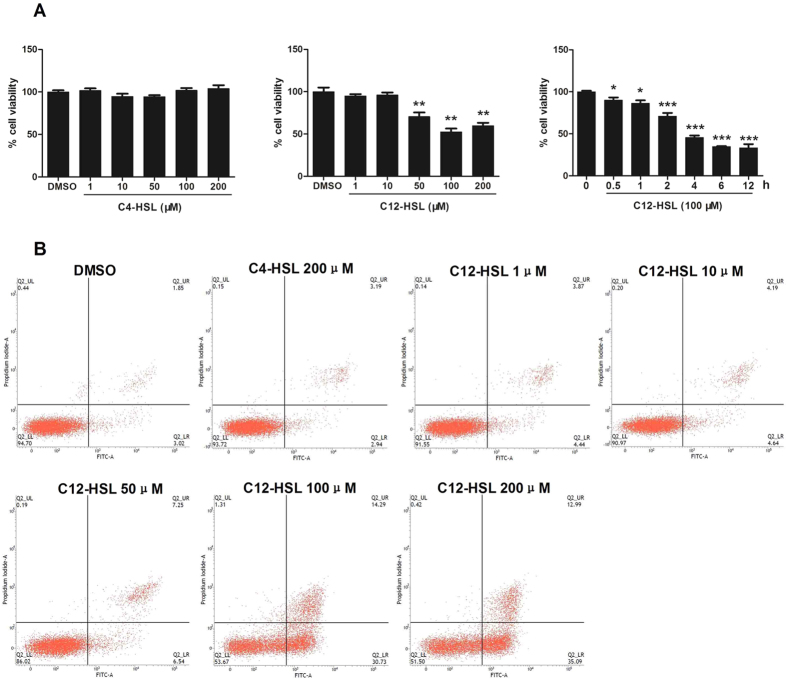
C12-HSL decreased cell viability in LS174T cells. (**A**) MTT assay of cell viability in AHLs treated LS174T cells and (**B**) flow cytometric analysis of apoptosis in AHLs-treated LS174T cells. Values are presented as mean ± SEM (n = 6) and expressed as percentage decrease in cell viability. *p < 0.05, **p < 0.01, ***p < 0.001 versus DMSO group.

AHLs vary greatly in carbon chain length, and this variation in carbon chain length may determine the distinct biological functions of AHLs^32^. To investigate the effect of AHL on the survival of human intestinal secretory cells and whether this effect is dependent on the length of the carbon chain of AHL, LS174T cells were treated with a long-carbon-chain AHL, C12-HSL, or a short-carbon-chain AHL, C4-HSL, at various concentrations, ranging from 0 to 200 μM, for 4 h. Our results show that treatment of LS174T cells with C12-HSL >50 μM significantly decreased cell viability (Fig. 1A). C4-HSL, however, did not affect the viability of LS174T cells (Fig. 1A). Additionally, cells treated with 100 μM of C12-HSL for different time periods showed time-dependent decreases in cell viability. To determine whether the survival inhibition of LS174T cells by C12-HSL was due to the induction of apoptosis, C12-HSL-treated LS174T cells were dual-stained with Annexin V and PI and analyzed by flow cytometry. The results indicated that C12-HSL treatment caused a dose-dependent increase in the apoptotic cell population. Consistent with the viability staining, C4-HSL (200 μM) did not affect the apoptosis of LS174T cells (Fig. 1B).

### C12-HSL induces mitochondrial damage in LS174T cells

**Figure 2:**
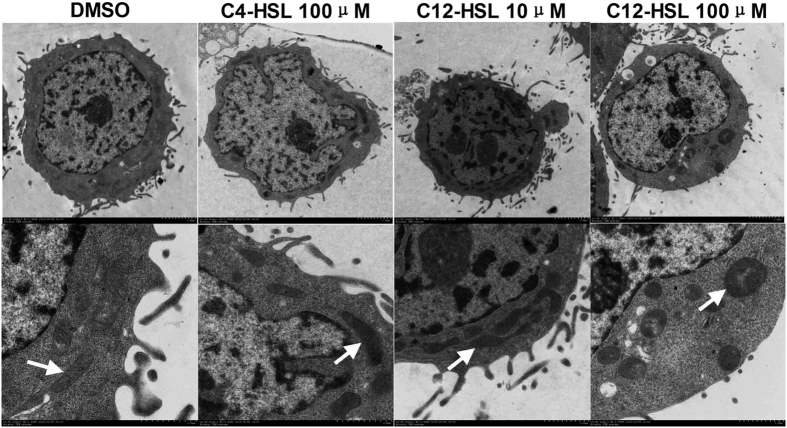
C12-HSL induced mitochondrial swelling in LS174T cells. LS174T cells from each group were processed for ultrastructure morphological evaluation. The arrow indicates the mitochondria.

Transmission electron microscopy was used to visualize the ultrastructural alterations in C4-HSL or C12-HSL-treated LS174T cells (Fig. 2). Cells treated with DMSO (control group) or C12-HSL at a low concentration (10 μM) displayed a normal subcellular structure. In contrast, cells treated with 100 μM C12-HSL for 4 h exhibited apparent mitochondrial swelling. Unsurprisingly, cells treated with 100 μM C4-HSL showed similar mitochondrial structure as the control cells.

### C12-HSL induces mitochondrial dysfunction in LS174T cells

**Figure 3:**
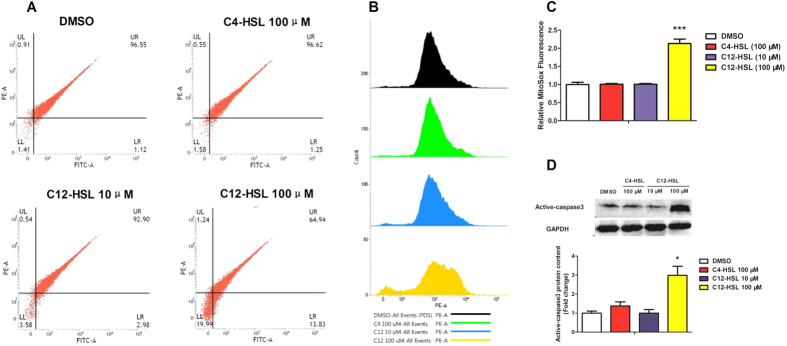
C12-HSL induces mitochondrial dysfunction in LS174T cells. (**A**) Flow cytometric analysis of mitochondrial membrane potential in LS174T cells treated with AHLs. (**B,C**) Flow cytometric analysis of mitochondrial ROS and (**D**) western blot showing protein expression of active-caspase3. Values are presented as mean ± SEM (n = 6) in (**C**) Mitosox Fluorescence and (**D**) protein content. *p < 0.05, ***p < 0.001 versus DMSO group.

**Figure 4:**
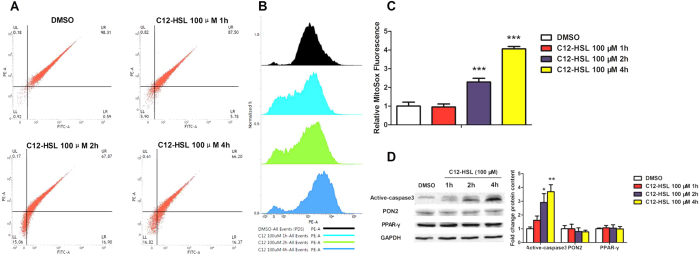
C12-HSL induces mitochondrial dysfunction and apoptosis for different time periods in LS174T cells. (**A**) Flow cytometric analysis of mitochondrial membrane potential in LS174T cells treated with C12-HSL for different time periods. (**B,C**) Flow cytometric analysis of mitochondrial ROS and (**D**) western blot showing protein expression of active-caspase3, PON2 and PPAR γ. Values are presented as mean ± SEM (n = 6) in (**C**) Mitosox Fluorescence and (**D**) protein content. *p < 0.05, **p < 0.01, ***p < 0.001 versus DMSO group.

Mitochondria play central roles in the regulation of apoptotic cell death, and loss of the mitochondrial membrane potential (ΔΨ_m_) and increased mitochondrial oxidative stress are profoundly associated with programmed cell death^33^. Therefore, we next examined the involvement of mitochondria in C12-HSL-induced apoptotic cell death by monitoring ΔΨ_m_ and oxidative stress in LS174T cells treated with C12-HSL. Using a fluorescent probe that specifically detects mitochondrial membrane potential (JC-1; Fig. 3A), we found that ΔΨ_m_ was significantly decreased in cells treated with 100 μM C12-HSL. In agreement with observed mitochondrial dysfunction as indicated by loss of ΔΨ_m_, treatment of LS174T cells with 100 μM C12-HSL also increased mitochondrial superoxide production (Fig. 3B,C). Additionally, compared to control cells, C12-HSL (100 μM) markedly increased active-caspase3 protein expression level (Fig. 3D). In contrast, C4-HSL at 100 μM and C12-HSL at 10 μM showed no effect on mitochondrial function in LS174T cells (Fig. 3). Otherwise, cells treated with 100 μM of C12-HSL for different time periods showed time-dependent change in ΔΨ_m_, mitochondrial superoxide production and active-caspase3 protein expression (Fig. 4). However, the PON2 and PPAR-γ protein levels did not showed changes in C12-HSL treated in LS174T cells (Fig. 4D). Collectively, these results suggested that mitochondrial dysfunction might be involved in C12-HSL-induced apoptosis in LS174T cells.

### C12-HSL inhibits secretion function of LS174T cells

**Figure 5:**
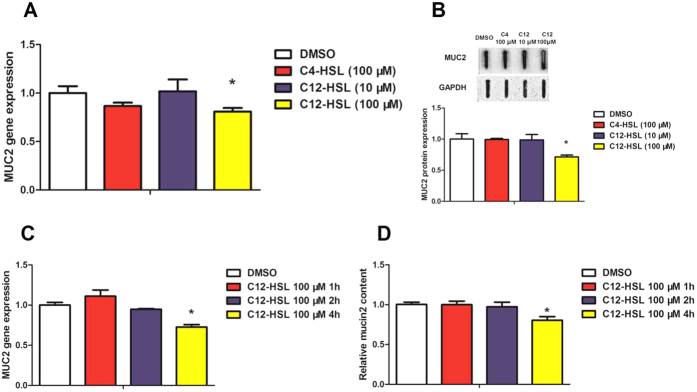
C12-HSL inhibited secretion function of LS174T cells. (**A**) Relative mRNA expression of MUC2. (**B**) Relative protein expression of MUC2. (**C**) Relative mRNA expression of MUC2 and (D) PAS assay of MUC2 content in culture medium. Values are presented as mean ± SEM (n = 6) in (**A–D**). *p < 0.05 versus DMSO group.

As shown in Fig. 5, the effects of AHLs on mucin production were next investigated in LS174T cells. C12-HSL at 100 μM significantly decreased MUC2 mRNA and protein expression levels compared to those of the DMSO control group. However, C4-HSL (100 μM) and C12-HSL (10 μM) did not significantly change MUC2 expression in LS174T cells. Additionally, we also tested the MUC2 expression in different time periods treated with 100 μM of C12-HSL. 100 μM of C12-HSL at 4 h markedly decreased the level of MUC2 in mRNA expression (Fig. 5C) and secreted to culture medium (Fig. 5D).

### Effects of lipid-raft disruptor MβCD on LS174T cells treated with C12-HSL

**Figure 6:**
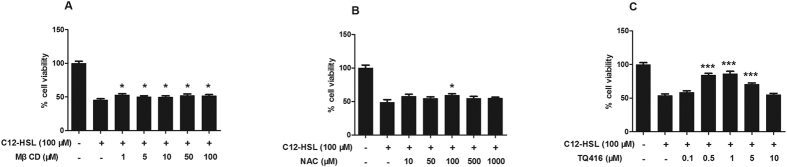
Effect of MβCD, NAC and TQ416 on cell viability in C12-HSL-treated LS174T cells. (**A**) MTT assay of cell viability in AHL- and MβCD-treated LS174T cells, (**B**) MTT assay of cell viability in AHLs- and NAC-treated LS174T cells and (**C**) MTT assay of cell viability in AHLs- and TQ416-treated LS174T cells. Values are presented as mean ± SEM (n = 6) and expressed as percentage increase in (**A**) MβCD, (**B**) NAC and (**C**) TQ416. *p < 0.05, ***p < 0.001 versus DMSO group.

**Figure 7:**
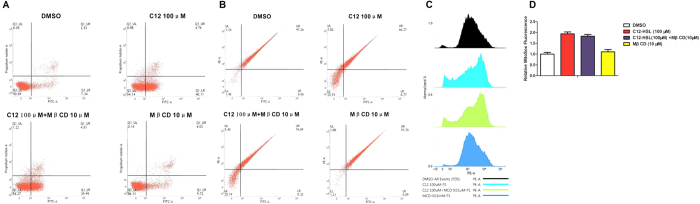
Effects of lipid-raft disruptor MβCD on LS174T cells treated with C12-HSL. (**A**) Flow cytometric analysis of apoptosis in MβCD and C12-HSL treated LS174T cells, (**B**) flow cytometric analysis of mitochondrial membrane potential and (**C,D**) flow cytometric analysis of mitochondrial ROS. Values are presented as mean ± SEM (n = 6) in (**D**) MitoSox Fluorescence.

Cholesterol in plasma membrane is a cellular receptor for AHLs^29^. To determine the functional importance of membrane cholesterol in mediating C12-HSL-caused cell damage, LS174T cells were cholesterol-depleted using the lipid raft sequester MβCD. Our results showed that treatment of LS174T cells with varying concentrations of MβCD (1–100 μM) marginally but significantly rescued C12-HSL-induced cell death in a dose-independent manner (Fig. 6A). Treatment of LS174T cells with 10 μM MβCD also slightly restored C12-HSL-caused decreases in ΔΨ_m_ (Fig. 7B), but did not influence C12-HSL-induced cell apoptosis or mitochondrial superoxide generation (Fig. 7A,C,D).

### Effects of oxidative stress inhibitor NAC on LS174T cells treated with C12-HSL

**Figure 8:**
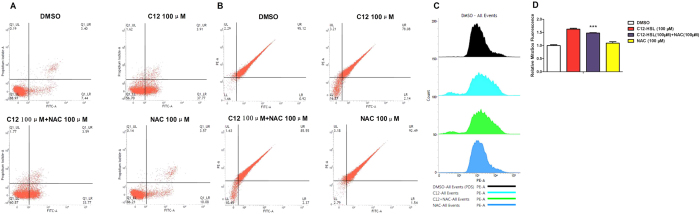
Effects of oxidative stress inhibitor NAC on LS174T cells treated with C12-HSL. (**A**) Flow cytometric analysis of apoptosis in NAC- and C12-HSL-treated LS174T cells, (**B**) flow cytometric analysis of mitochondrial membrane potential and (**C,D**) flow cytometric analysis result of mitochondrial ROS. Values are presented as mean ± SEM (n = 6) in (**D**) MitoSox Fluorescence. ***p < 0.001 versus C12-HSL treatment group.

To determine whether oxidative stress mediates C12-HSL-induced LS174T cells damage, we tested the effect of the antioxidant NAC on C12-HSL-induced cell apoptosis and mitochondrial impairment. Treatment with NAC at 100 μM, but not at other tested concentrations, showed a protective effect and rescued C12-HSL-induced cell death (Fig. 6B). Additionally, NAC (100 μM) significantly decreased mitochondrial ROS generation (Fig. 8C,D) but had only a marginal effect on the C12-HSL-induced decrease in ΔΨ_m_ and increase in cell apoptosis in LS174T cells (Fig. 8A,B).

### Effects of PON2 inhibitor TQ416 on LS174T cells treated with C12-HSL

**Figure 9:**
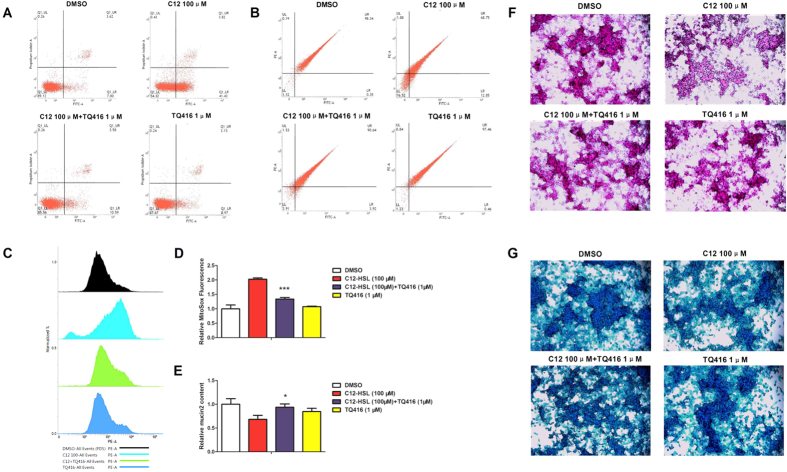
Effects of PON2 inhibitor TQ416 on LS174T cells treated with C12-HSL. (**A**) Flow cytometric analysis of apoptosis in TQ416- and C12-HSL-treated LS174T cells, (**B**) flow cytometric analysis of mitochondrial membrane potential, (**C,D**) flow cytometric analysis of mitochondrial ROS, (**E**) PAS assay of MUC2 content in LS174T cells, (**F**) PAS staining and (**G**) Alcian blue staining. Values are presented as mean ± SEM (n = 6) in (**D**) MitoSox Fluorescence and (**E**) MUC2 content. *p < 0.05, ***p < 0.001 versus the C12-HSL treatment group.

Paraoxonase 2 (PON2) catalyzes the hydrolysis of C12-HSL and promotes C12-induced apoptosis^34^. To investigate the functional relevance of PON2 hydrolase in C12-HSL induction of cell damage, the PON2 inhibitor TQ416 was used to inhibit PON2 activity. When used at a concentration range of 0.5–5 μM, TQ416 remarkably rescued C12-HSL-induced cell death. The strongest rescuing effect of TQ416 was observed at 1 μM but not at other concentration (Fig. 6C). This result was consistent with previous study^27^, and this concentration was used in the following experiments. Further analysis demonstrated that TQ416 significantly reversed C12-HSL-induced apoptotic cell death (Fig. 9A), restored the C12-HSL-caused decrease in ΔΨ_m_ (Fig. 9B), and inhibited mitochondrial ROS generation (Fig. 9C,D) in LS174T cells. These results suggest that hydrolysis of PON2 may mediate the severe damage caused by C12-HSL in LS174T cells.

Next, the intracellular MUC2 level was measured in LS174T cells by PAS assay. Treatment with C12-HSL at 100 μM for 4 h markedly decreased MUC2 production in LS174T cells and TQ416 (1 μM) completely restored this decrease (Fig. 9E). Next, PAS and alcian blue staining were performed to evaluate the mucous glycoprotein and sulfation level. The mucous glycoprotein and sulfation level were obviously reduced in C12-HSL-treated LS174T cells, but treatment with TQ416 restored the mucous glycoprotein and sulfation level (Fig. 9F,G). These results imply that TQ416 may be a potent inhibitor of C12-HSL-induced cellular responses in LS174T.

### C12-HSL induces pro-inflammatory cytokine expression in LS174T cells

**Figure 10:**
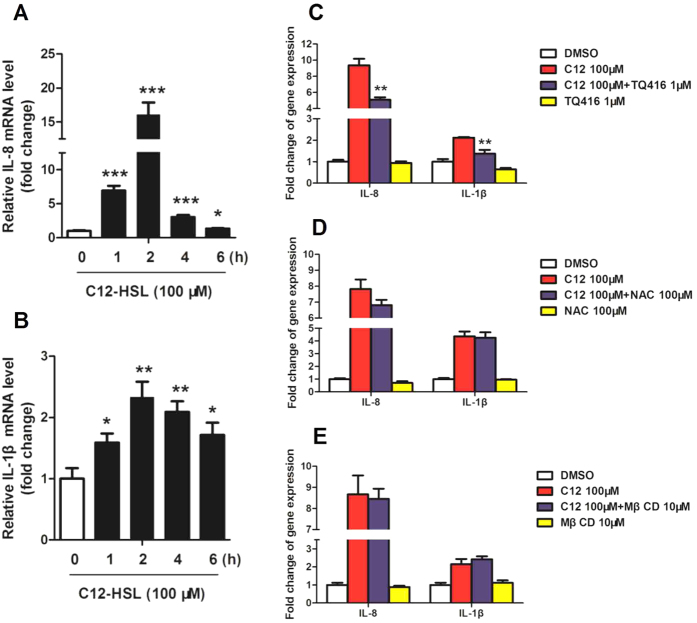
C12-HSL induces pro-inflammatory cytokine expression in LS174T cells. (**A**) Relative mRNA expression of IL-8. (**B**) Relative mRNA expression of IL-1β. (**C–E**) Relative mRNA expression of IL-8 and IL-1β. Values are presented as mean ± SEM (n = 6) in (**A–E**). *p < 0.05, **p < 0.01, ***p < 0.001 versus DMSO group in (**A,B**). **p < 0.01 versus the C12-HSL treatment group in (**C**).

As shown in Fig. 10, the effects of AHLs on pro-inflammatory cytokine production were next investigated in LS174T cells. The mRNA expression of IL-8 and IL-1β were significantly up-regulated from 1 h after incubation with 100 μM C12-HSL (Fig. 10A,B). We also explored the ability of MβCD, NAC and TQ416 to regulate IL-8 and IL-1β production. Unsurprisingly, TQ416 significantly attenuated the production of IL-8 and IL-1β compared with the C12-HSL alone in LS174T cells (Fig. 10C). However, MβCD and NAC did not affect the production of IL-8 and IL-1β of LS174T cells (Fig. 10D,E).

### Timing effect of TQ416 administration on C12-HSL-induced cell death

**Figure 11:**
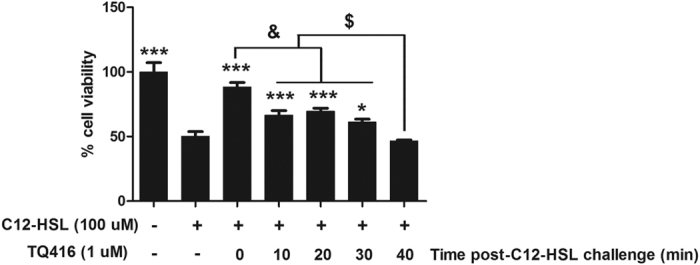
Timing effect of TQ416 administration on C12-HSL-induced cell death. MTT assay of cell viability in TQ416 and C12-HSL treated LS174T cells. Values are presented as mean ± SEM (n = 6) and expressed as percentage. *p < 0.05, ***p < 0.001 versus C12-HSL treatment group. ^&^p < 0.001 versus TQ416 post-C12-HSL treatment 0 min group. ^$^p < 0.001 versus TQ416 post-C12-HSL treatment 40 min group.

To determine the effect of exposure timing of TQ416 to prevent C12-HSL-induced cell death, TQ416 was added to cultures together with C12-HSL simultaneously or 10, 20, 30, or 40 min after the addition of C12-HSL. When added with C12-HSL, TQ416 nearly completely blocked C12-HSL-induced cell death. However, this rescuing effect of TQ416 significantly decreased when TQ416 was administered 10–30 min post-C12-HSL challenge and was completely lost when TQ416 was administered 40 min later (Fig. 11). These results indicate that the rescuing effect of TQ416 on C12-HSL-induced cell death is dependent on the timing of TQ416 administration.

### C12-HSL decreases cell viability and induces mucin abnormal expression of HCT116 cells

**Figure 12:**
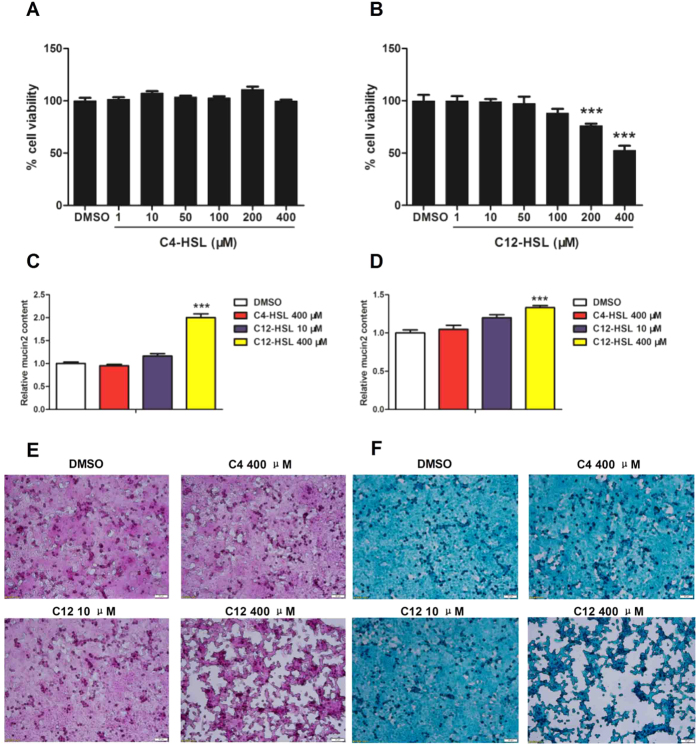
C12-HSL decreases cell viability and induces mucin abnormal expression of HCT116 cells. (**A**) MTT assay of cell viability in C4-HSL treated HCT116 cells. (**B**) MTT assay of cell viability in C12-HSL treated HCT116 cells. (**C**) PAS assay of MUC2 content in HCT116 cells. (**D**) PAS assay of MUC2 content in culture medium. (**E**) PAS staining and (**F**) Alcian blue staining. Values are presented as mean ± SEM (n = 6) in (**A–D**). ***p < 0.001 versus DMSO group.

To determine whether AHLs mediates cell viability and mucus secretion of colonic epithelial cell lines, HCT116 cells were treated with C4-HSL or C12-HSL, at various concentrations, ranging from 0 to 400 μM, for 4 h. Our results show that treatment of HCT116 cells with C12-HSL >200 μM significantly decreased cell viability in a dose-dependent manner (Fig. 12B). C4-HSL, however, did not affect the viability of HCT116 cells (Fig. 12A). The effects of AHLs on mucin production were next investigated in HCT116 cells. C12-HSL at 400 μM significantly increased MUC2 levels of intracellular (Fig. 12C) and secreted to culture medium (Fig. 12D) compared to those of the DMSO control group. However, C4-HSL (400 μM) and C12-HSL (10 μM) did not significantly change MUC2 expression in HCT116 cells (Fig. 12C,D). Next, PAS and alcian blue staining were performed to evaluate the mucous glycoprotein and sulfation level. The mucous glycoprotein and sulfation level were obviously increased in C12-HSL (400 μM)-treated HCT116 cells, but treatment with C4-HSL (400 μM) and C12-HSL (10 μM) did not change the mucous glycoprotein and sulfation level (Fig. 12E,F).

## Discussion

MUC2, a glycoprotein synthesized by goblet cells, is the most abundant mucin covering the outer and inner intestinal epithelium^35^. Intestinal infections caused by bacteria, viruses, and parasites alter goblet cells response and mucin production^36^. Alterations in colonic mucin biochemistry, including decreased oligosaccharide chain length and reduced sulfation have been observed in patients with inflammatory bowel disease^37^. QS molecules produced by bacteria in the gut are associated with perturbation of host epithelial cell homeostasis and development of intestinal diseases^3^. Many studies have reported the detrimental effects of AHLs produced by gram-negative bacteria on intestinal epithelial barrier function, inflammation, and cell migration^23^,^30^,^31^. However, the biological effects of AHLs on goblet cells were unclear. In this study, we evaluated the effects of two representative long acyl chain (C12-HSL) and short-chain (C4-HSL) AHLs on cell viability and secretory function of goblet cell line LS174T. We found that exposure to C12-HSL, but not C4-HSL, perturbed LS174T cells viability and induced a high level of cell apoptosis. This is consistent with other studies conducted in the epithelial cell line Caco-2 cell^31^,^38^,^39^. However, both C4-HSL and C12-HSL were reported to induce apoptosis and barrier disruption by promoting expression of inflammatory cytokines in different types of cells^17^,^23^,^30^,^39^,^40^,^41^. These inconsistent results suggest the regulatory effects of AHLs on host cells are cell-type dependent and AHL-specific.

Although the molecular mechanisms involved in C12-HSL triggered apoptosis have not been determined and the goal of identifying AHLs receptors in mammalian cells is still in the early stages, some signaling pathways and an intracellular rather than a cell surface receptor mechanism for mediation of AHLs activity have been described^30^,^42^,^43^. Due to its lipophilicity, C12-HSL can rapidly enter mammalian cells^16^. Previous studies demonstrated that C12-HSL triggers apoptosis in multiple cell types, including gut epithelial cells, airway epithelial cells, breast carcinoma cells, macrophages, and neutrophils^17^,^18^,^19^,^20^,^21^,^22^. These responses of C12-HSL on host cells may be guided by different mechanisms; at higher C12-HSL concentrations >25 μM, intracellular events, such as acidification by PON2, may predominate, but for C12-HSL at <10 μM, more sensitive receptor driven effects will predominate^34^. C12-HSL at relatively low concentrations (between 10 to 30 μM) can reduce viability accompanied by apoptosis via the suppression of AKT phosphorylation in undifferentiated Caco-2 cells^38^. This effective concentration of C12-HSL to trigger apoptosis in intestinal epithelial cells is lower than the levels measured in biofilms^44^. However, in this study, we did not observe pro-apoptotic effect in LS174T goblet cells treated with C12-HSL at concentrations less than 25 μM (data not shown). Additionally, there was no significant change in MUC2 expression and sulfation after treatment with 10 μM C12-HSL. Again, these results suggest cell-specific effects of C12-HSL in host cells.

Paraoxonase 2 (PON2) expressed intracellularly is found widely in many mammalian tissues and cell types and efficiently hydrolyzes C12-HSL to C12-HSL-acid^45^,^46^,^47^. PON2 may be a central regulator of host cell responses to C12-HSL via intracellular acidification to mediate subsequent biological responses such as calcium release and stress signaling^34^. In our study, although the PON2 protein level did not showed changes in C12-HSL treated in LS174T cells, its high expression abundance enough to complete the acidification. Our results showed that TQ416, a PON2 enzyme inhibitor, significantly restored LS174T cells viability by preventing ROS production and cell apoptosis when administered together with C12-HSL. Moreover, TQ416 significantly attenuated the production of pro-inflammatory cytokine (IL-8 and IL-1β) compared with the C12-HSL alone in LS174T cells. C12-HSL was reported to rapidly trigger multiple events associated with apoptosis in mouse embryo fibroblasts, accompanied by the depolarization of mitochondrial membrane potential, observed at 5 min and completed within 20 min^34^,^48^. Consistently, when LS174T cells were treated with TQ416 10 mins after C12-HSL exposure, the restorative effect was significantly decreased relative to that seen with simultaneous treatment and the rescue effect was eliminated when TQ416 was treated 40 mins after C12-HSL exposure. Taken together, our results suggest that C12-HSL rapidly (within 40 mins) hydrolyzes to C12-HSL-acid by PON2 in LS174T cells, and then triggers a series of biological effects including oxidative stress, apoptotic, and altered mucous glycoprotein and sulfation.

Eum *et al*., reported that Methyl-β-cyclodextrin (MβCD), which is used to remove cholesterol from cultured cell and is used to remove lipid rafts, effectively blocked the increase in C12-HSL-induced permeability across Caco-2 monolayers^29^. Cholesterol in plasma membrane has even been suggested as a cellular receptor of AHLs^29^. However, in this study, MβCD only showed a marginal effect on countering the damage of cell viability of LS174T induced by C12-HSL. It is well-documented that C12-HSL enters mammalian cells by passive mechanisms and that there is no preferential membrane association^49^. Consistently, we found no evidence for the involvement of lipid rafts in mediating the cell damage processes in C12-HSL-treated LS174T cells.

We also employed NAC, a powerful antioxidant component, to investigate the importance of oxidative stress in the process of C12-HSL-mediated damage to LS174T cells. A previous report demonstrated that the NAC did not inhibit quorum sensing^50^. Similarly, in our study, although NAC significantly decreased mitochondrial ROS generation, there was no obvious restorative effect of NAC on cells damage induced by C12-HSL in LS174T cells. These results suggest the acidification of LS174T cells induced by PON2 is the initial step of apoptosis. After formation, C12-HSL-acid accumulated in cells, rapidly leading to oxidative stress and apoptosis, and NAC is unable to prevent this rapid process.

As mentioned above, the regulatory effects of AHLs on host cells are cell-type dependent. We also employed HCT116 cells, a common colonic epithelial cell line, to investigate the effect of AHLs on cell viability and mucus secretion of colonic epithelium. Interestingly, we found that exposure to C12-HSL (>200 μM), disturbed HCT116 cells viability. This result suggests the goblet LS174T cells were more vulnerable in face of C12-HSL. Previous study reported that the *Pa* autoinducer C12-HSL contributes to excessive mucin production in chronic bacterial infection^51^. Consistent with this report, in the present study, we discovered that the levels of MUC2 protein and mucous glycoprotein were dramatically elevated after incubation with high concentration C12-HSL (400 μM). These results suggest that although C12-HSL induced the decreased of cell viability and abnormality of mucus expression in LS174T and HCT116 cells, the goblet LS174T cells more sensitive to C12-HSL.

A major conclusion from this study is that C4-HSL and low concentrations of C12-HSL showed no effects on cell viability and mucin secretion in goblet LS174T cells, but C12-HSL at high concentration (100 μM) rapidly triggers events associated with the intrinsic pathway leading to apoptosis: mitochondrial swelling, ΔΨ_m_ depolarization, enhanced mitochondrial ROS generation, and activation of caspase3. The inhibitor of PON2 enzyme TQ416, but not the lipid-raft disruptor MβCD or oxidative stress inhibitor NAC, can rescue the effects of C12-HSL on cell viability, apoptosis, and the secretion function of goblet LS174T cells.

## Materials and Methods

### Chemicals

C12-HSL and C4-HSL were purchased from Sigma-Aldrich (St. Louis, MO) and their stock solutions (100 mM) were prepared in dimethyl sulfoxide (DMSO). Anti-active-caspase3 antibody, anti-MUC2 antibody, anti-PON2 antibody, anti-PPAR γ antibody, anti-GAPDH antibody, and horseradishperoxidase-conjugated secondary antibodies were obtained from Santa Cruz Biotechnology (Santa Cruz, CA). Methyl-β-cyclodextrin (MβCD) and N-acetyl-L-cysteine (NAC) were purchased from Sigma-Aldrich (St. Louis, MO). Triazolo[4,3-*a*]quinolone (TQ416) was purchased from ChemDiv (San Diego, USA). The concentrations of all of tested pharmacological inhibitors did not show any significant cytotoxic effects by themselves as confirmed by FACS analysis in each experiment.

### Cells

The LS174T cell line (ATCC CL-188) is a human colon cancer cell line that exhibits characteristics of normal colonic mucosal cells, including microvilli prominent in secretory cells and the presence of intracytoplasmic mucin vacuoles. The HCT116 cell line (ATCC CCL -247) is a human colon cancer cell line. LS174T and HCT116 cells were grown at 37 °C in 5% CO_2_ in RPMI 1640 supplemented with 10% FBS and antibiotics (10 U/ml penicillin G and 10 mg/ml streptomycin). In all the assays, vehicle control (DMSO) was found to be non-toxic to LS174T and HCT116 cells and did not induce either apoptosis or oxidative stress to LS174T cells.

### Cell viability assay

Cell viability was determined using the conversion of MTT to formazan via mitochondrial oxidation. Cells were pretreated with the indicated inhibitors prior to C12-HSL exposure for various times. MTT solution was then added to each well at a final concentration of 1 mg/ml per well and the plates were incubated at 37 °C for another 2 h. After incubation, 150 μl DMSO was added to each well to dissolve the formed formazan and the absorbance was recorded at 570 nm.

### Transmission electron microscopy

The cells of four groups were fixed with 2.5% (v/v) glutaraldehyde in PBS and post-fixed with 1.0% (w/v) osmium tetroxide in the same buffer, followed by dehydration with a graded series of ethanol. This was followd by propyleneoxide treatment and then the cells were embedded in epoxy resin and sectioned. The ultrathin sections were contrasted with ethanolic uranyl acetate and lead citrate and observed under a transmission electron microscope (JEOLJEM-1210, Japan).

### Flow cytometry

LS174T cells apoptosis status was detected with an Annexin V and propidium iodide (PI) staining kit (BD Biosciences) according to the manufacturer’s instructions. Briefly, the cells were detached with 0.05% trypsin/EDTA and 1 × 10^5^ cells were resuspended with annexin V binding buffer. The cells were then stained with annexin V (25 μg/ml) and PI (125 ng/ml) and incubated for 15 min at room temperature in the dark. The sample was analysed using FACSVerse flow cytometer (BD Biosciences, USA).

The JC-1 staining kit (BD Biosciences) was used to detect changes in the mitochondrial membrane potential (ΔΨ_m_) according to the manufacturer’s instructions. Briefly, after the culture medium was removed, the cells were washed three times with PBS. After dilution to a final concentration of 2 μM with serum-free RPMI 1640, JC-1 was added to the cells and incubated for 20 min at 37 °C. Next, cells were washed three times with PBS. The cells were resuspended in PBS and the fluorescence intensity was measured for more than 10,000 cells of each sample by flow cytometry (FACSVerse).

The intracellular oxidant levels in LS174T cells were measured with MitoSox red mitochondrial superoxide indicator (Invitrogen) as described previously^52^. Briefly, after the culture medium was removed, the cells were washed three times with PBS. MitoSox red mitochondrial superoxide indicator, diluted to a final concentration of 4 mM with serum-free RPMI 1640, was added to the cells and incubated for 20 min at 37 °C in the dark. The cells were then washed three times with PBS. The cells were resuspended in PBS and the fluorescence was measured immediately by FACSVerse flow cytometer. The level of intracellular oxidant levels corresponded with an increase in fluorescence and was calculated as the percentage of the measured signals for control cells.

### RNA extraction, reverse transcription and real-time quantitative PCR

Messenger RNA extraction and reverse transcription were conducted using SuperScript III First-Strand Synthesis System (Invitrogen, USA), according to the manufacturer’s protocol. The synthesized cDNA was used for quantitative real-time PCR. Real-time PCR was performed with Mx3000P (Stratagene, USA). The 2^−ΔΔCt^ method was used to analyze real-time PCR data. Expression of mRNA was investigated using the following primers: 5′-CAGCACCGATTGCTGAGTTG-3′ and 5′-GCTGGTCATCTCAATGGCAG-3′ for MUC2; 5′- AGCTGGCCGTGGCTCTCT-3′ and 5′- CTGACATCTAAGTTCTTTAGCACTCCTT-3′ for IL-8; 5′-GAAATGATGGCTTATTACAGTGGC-3′ and 5′-GCTGTAGTGGTGGTCGGAGATT-3′ for IL-1β; 5′-TGCACCACCAACTGCTTAGC-3′and 5′-GGCATGGACTGTGGTCATGAG-3′ for GAPDH. All data were normalized against the house-keeping gene GAPDH and expressed as the fold difference relative to the mean of relevant control samples.

### Preparation of cellular lysates for Western Blot analysis

LS174T cells were solubilized in cell lysis buffer containing 1%Triton X-100, 10 mM Tris (pH 7.4), 1 mM EDTA, 1 mM EGTA,150 mM NaCl, and a proteinase inhibitor mixture (Roche Applied Science) and incubated for 1 h on ice. The scraped suspensions were centrifuged at 14,000 rpm for 15 min at 4 °C, and the protein concentration was determined using a BCA protein assay kit (Pierce Thermo Scientific). After denaturation by boiling for 5 min, 40 μg of protein was separated by 15% SDS-PAGE, transferred on to nitrocellulose membrane (BioTrace, Pall Co, USA), blocked with 5% BSA in Tris buffer (pH 7.5) with 0.1% Tween 20 for 2 h, then incubated overnight at 4 °C with the anti-active-caspase3 antibody. Then the blots were incubated with the relevant second antibody for 2 h at 25 °C. Finally, the blot was washed and detected by enhanced chemiluminescence’s (ECL) using the LumiGlo substrate (Super Signal West Pico Trial Kit, Pierce, USA), and the signals were recorded by an imaging System (Bio-Rad, USA), and analyzed with Quantity One software (Bio-Rad, USA). GAPDH was used as a loading control for the Western blot. The protein content was expressed as the fold change relative to the mean value of the control group.

### Mucin protein assay

MUC2 mucin proteins were measured by modification of a previously reported method^53^. Briefly, the LS174T cells were precultured overnight in a 6-well plate, and then cultured for an additional 3 days with different treatments. After removal of the medium, the cells were lysed and the protein concentration was determined using a BCA protein assay kit (Pierce Thermo Scientific), and 16 μg of protein was then blotted onto nitrocellulose membrane (BioTrace, Pall Co, USA). Membranes were incubated with 5% skimmed milk for blocking, and then the anti-MUC2 antibody, followed by incubation with the relevant second antibody. GAPDH was used as an internal control. Image capture and data analysis was performed as described for the western blot.

### PAS assay

LS174T cells were disrupted in PBS using sonication (Sonics VCX105, USA) to obtain soluble proteins. Protein concentration was determined using a BCA protein assay kit. All samples were diluted to the same concentration. The mucous glycoprotein in soluble fractions was measured as previously reported^54^. Briefly, cellular soluble fractions and culture medium were incubated with 0.1% periodic acid (Sigma-Aldrich) for 2 h at room temperature. Next, the Schiff reagent (Sigma-Aldrich) was added and incubated for 30 min at room temperature. The OD of the resulting solution at 550 nm wavelength was taken as a measure of the amount of PAS-positive product present. The PAS OD value was expressed as the fold change relative to the mean value of control group.

### PAS and alcian blue staining

LS174T cells were fixed in 4% paraformaldehyde at 4 °C overnight and stained using a PAS kit (Sigma-Aldrich) and alcian blue solution (Sigma-Aldrich), according to the manufacturer’s instructions.

### Statistical analysis

Data are presented as means ± SEM. The data were tested for normal distribution and statistical significance was assessed by the independent sample t-test using SPSS (SPSS version 11.0 for Windows; SPSS Inc., Chicago, IL, USA) software packages. Data were considered statistically significant when *P* < 0.05.

## Additional Information

**How to cite this article**: Tao, S. *et al*. Paraoxonase 2 modulates a proapoptotic function in LS174T cells in response to quorum sensing molecule N-(3-oxododecanoyl)-L-homoserine lactone. *Sci. Rep.* **6**, 28778; doi: 10.1038/srep28778 (2016).
